# Integrative Analysis of a Novel Eleven-Small Nucleolar RNA Prognostic Signature in Patients With Lower Grade Glioma

**DOI:** 10.3389/fonc.2021.650828

**Published:** 2021-06-07

**Authors:** Teng Deng, Yizhen Gong, Xiwen Liao, Xiangkun Wang, Xin Zhou, Guangzhi Zhu, Ligen Mo

**Affiliations:** ^1^ Department of Neurosurgery, Guangxi Medical University Cancer Hospital, Nanning, China; ^2^ Evidence-based Medicine Teaching and Research Section, Affiliated Hospital of Guilin Medical University, Guilin, China; ^3^ Department of Hepatobiliary Surgery, The First Affiliated Hospital of Guangxi Medical University, Nanning, China

**Keywords:** small nucleolar RNA, lower grade glioma, The Cancer Genome Atlas, molecular mechanism, immune infiltration

## Abstract

**Objective:**

The present study used the RNA sequencing (RNA-seq) dataset to identify prognostic snoRNAs and construct a prognostic signature of The Cancer Genome Atla (TCGA) lower grade glioma (LGG) cohort, and comprehensive analysis of this signature.

**Methods:**

RNA-seq dataset of 488 patients from TCGA LGG cohort were included in this study. Comprehensive analysis including function enrichment, gene set enrichment analysis (GSEA), immune infiltration, cancer immune microenvironment, and connectivity map (CMap) were used to evaluate the snoRNAs prognostic signature.

**Results:**

We identified 21 LGG prognostic snoRNAs and constructed a novel eleven-snoRNA prognostic signature for LGG patients. Survival analysis suggests that this signature is an independent prognostic risk factor for LGG, and the prognosis of LGG patients with a high-risk phenotype is poor (adjusted P = 0.003, adjusted hazard ratio = 2.076, 95% confidence interval = 1.290–3.340). GSEA and functional enrichment analysis suggest that this signature may be involved in the following biological processes and signaling pathways: such as cell cycle, Wnt, mitogen-activated protein kinase, janus kinase/signal transducer and activator of tran-ions, T cell receptor, nuclear factor-kappa B signaling pathway. CMap analysis screened out ten targeted therapy drugs for this signature: 15-delta prostaglandin J2, MG-262, vorinostat, 5155877, puromycin, anisomycin, withaferin A, ciclopirox, chloropyrazine and megestrol. We also found that high- and low-risk score phenotypes of LGG patients have significant differences in immune infiltration and cancer immune microenvironment.

**Conclusions:**

The present study identified a novel eleven-snoRNA prognostic signature of LGG and performed a integrative analysis of its molecular mechanisms and relationship with tumor immunity.

## Introduction

Tumors derived from the neuroepithelium are collectively called gliomas and are the most common malignant primary intracranial tumor (1). Lower-grade gliomas are well-differentiated gliomas, and their preferred treatment is surgery, followed by radiotherapy, chemotherapy, immunotherapy, and targeted therapy. Just like any other cancers, glioma is also due to the interaction of innate genetic risk factors and environmental carcinogenic factors (e.g., radiation exposure). Some known genetic disease, such as neurofibromatosis (type I), is genetic susceptibility factor for glioma (2). With the development of high-throughput sequencing technology, we have found that more and more genetic variants are connected with the occurrence, development and clinical outcome of cancers. TCGA is a database resource for whole-genome multi-omics sequencing of 33 human cancers based on high-throughput sequencing technology (3). It can provide the whole genome multi-omics data set of glioma, so that we can further explore its genetic variation (4).

SnoRNA belong to a small non-coding RNA, mostly enriched in nucleolus (5). SnoRNA plays an important role in the splicing processing and post-transcriptional modification of ribosomal precursor RNA. Previous research reports suggested that snoRNAs were dysregulated between glioma tumor and adjacent non-tumor tissues, and involved in the regulation of cancer cell apoptosis, proliferation and invasion, including in glioma (6–8). However, there are few reports on the prognostic value of snoRNA in gliomas (8). We found that there is no research report on screening LGG prognostic snoRNAs based on genome-wide dataset. To fill this gap, we designed and implemented this study. The present study was used the RNA sequencing (RNA-seq) dataset to screen prognostic-related snoRNAs and construct a prognostic signature for The Cancer Genome Atla (TCGA) lower grade glioma (LGG) cohort, and conduct a comprehensive investigation of its molecular mechanisms and relationship with tumor immunity.

## Materials and Methods

### Data Source

The original level 3 raw RNA sequencing (RNA-seq) dataset of lower grade glioma used in our study comes from TCGA data portal (4). Clinical parameters of TCGA LGG cohort were obtained from UCSC Xena (http://xena.ucsc.edu) (9). We obtained 940 snoRNAs from TCGA RNA-seq dataset into the current analysis. The original HTSeq-Counts RNA-seq data set was normalized in the R platform through the edgeR package (10). We eliminated snoRNAs with a mean value less than 1, and obtained a total of 137 snoRNAs for subsequent survival analysis. We obtained a total of 529 RNA-seq data sets of 511 patients from the TCGA data portal, and we excluded 18 recurrent cancer tissues. Of the 511 patients, we excluded five patients whose survival time was 0 or who had no survival time. We also excluded 18 patients who had a history of other malignancies or who received radiotherapy or chemotherapy before surgery. Finally, 488 patients were recruited for the subsequent comprehensive analysis, which included survival analysis and functional mechanism analysis. IDH1 mutation data of TCGA LGG cohort were obtained from cBioPortal for Cancer Genomics (https://www.cbioportal.org/index.do).

### Survival Analysis of snoRNAs Signature

Prognostic snoRNAs screening was performed on the R platform using *survival* packet and univariate Cox proportional hazards regression model. The prognostic snoRNAs signature is constructed in the R platform using the step function. We use prognostic-related snoRNAs as variables into the multivariate Cox proportional hazard regression model, and the Cox regression coefficient (β) as the weight of each snoRNAs included in the signature to compose the risk score. The snoRNAs signature calculation formula was as follows: risk score = Exp of snoRNA_1_ × β_1_snoRNA_1_ + Exp of snoRNA_2_ × β_2_snoRNA_2_ + Exp of snoRNA_n_ × β_n_snoRNA_n_ (Exp: expression) (11–13). Subsequently, we also used the time-dependent ROC curve, nomogram, and combined effect survival analysis to conduct a comprehensive analysis of the prognostic value of this risk score signature in LGG. The nomogram was drawn in the R platform using the *rms package.* The time-dependent ROC curve was performed in the R platform using the survival ROC package. The combined effect survival analysis was performed in SPSS version 22.0 using the multivariate Cox proportional hazards regression model.

### Functional Enrichment Analysis of snoRNAs Signature Based on Whole-Gene RNA-seq Dataset

SnoRNA is a small non-coding RNA encoded by introns, and its function is exerted through the regulation of protein-coding genes (PCGs). We use the *Cor* function and whole genome encoded RNA-seq dataset of LGG tumor tissues samples to screen the PCGs that related to snoRNAs, were performed on the R platform. We identified that the absolute value of Pearson correlation coefficient (r) between snoRNAs and genes were greater than 0.4 and P <0.05 considered to be the co-expression PCGs of snoRNA. The normalization of PCGs’ RNA-seq dataset was also performed by *edgeR*. Then, we use the Database for Annotation, Visualization and Integrated Discovery v6.8 (DAVID v6.8, https://david.ncifcrf.gov/home.jsp) online tool to perform functional annotations on the snoRNAs we have screened through the snoRNA co-expression PCGs (14). In addition, we also used the gene set enrichment analysis (GSEA, http://software.broadinstitute.org/gsea/index.jsp) desktop installer to perform functional enrichment analysis between high-risk and low-risk phenotypes for purpose of further investigate the molecular mechanisms of prognostic differences between this two phenotypes (15). For further investigate the molecular mechanism, we further used *edgeR* to screen differentially expressed genes (DEGs) between high-risk and low-risk phenotypes. Then we use DAVID v6.8 to annotate the functions of these DEGs. We subsequently used these DEGs and connectivity map (CMap, https://portals.broadinstitute.org/cmap/) online tool to identify potential drugs for LGG patients with a high-risk phenotype (16, 17). PubChem (https://pubchem.ncbi.nlm.nih.gov) and STITCH (http://stitch.embl.de/cgi/) online tools were applied to obtain the chemical structure of drugs and the drug-gene interaction networks, respectively (18, 19).

### Tumor Immune Infiltration and Microenvironment Analysis

CIBERSORT package was used to compute the abundance and scale of 22 immune cells in LGG tumor tissues to assess the degree of immune infiltration of LGG tumor samples (20, 21). We use the ESTIMATE (Estimation of STromal and Immune cells in MAlignant Tumor tissues using Expression data) package and LGG’s whole genome RNA-seq dataset to calculate the tumor immune microenvironment on the R platform, including the values of immune and stromal cells (22).

### Statistical Analysis

The calculation of FDR adopts Benjamini–Hochberg method (23). Survival analysis uses log-rank test of Kaplan–Meier algorithm, as well as univariate and multivariate Cox proportional hazards regression models. Clinical parameters with P <0.05 were included in multivariate Cox proportional hazards regression model for adjustment. Survival curves, heat maps and volcano maps were drawn on the R platform using the *ggplot2* package. P <0.05 identified the difference to reach statistical significance. All statistics are computed by SPSS version 22.0.

## Results

### Survival Analysis of snoRNAs Signature

**Figure 1:**
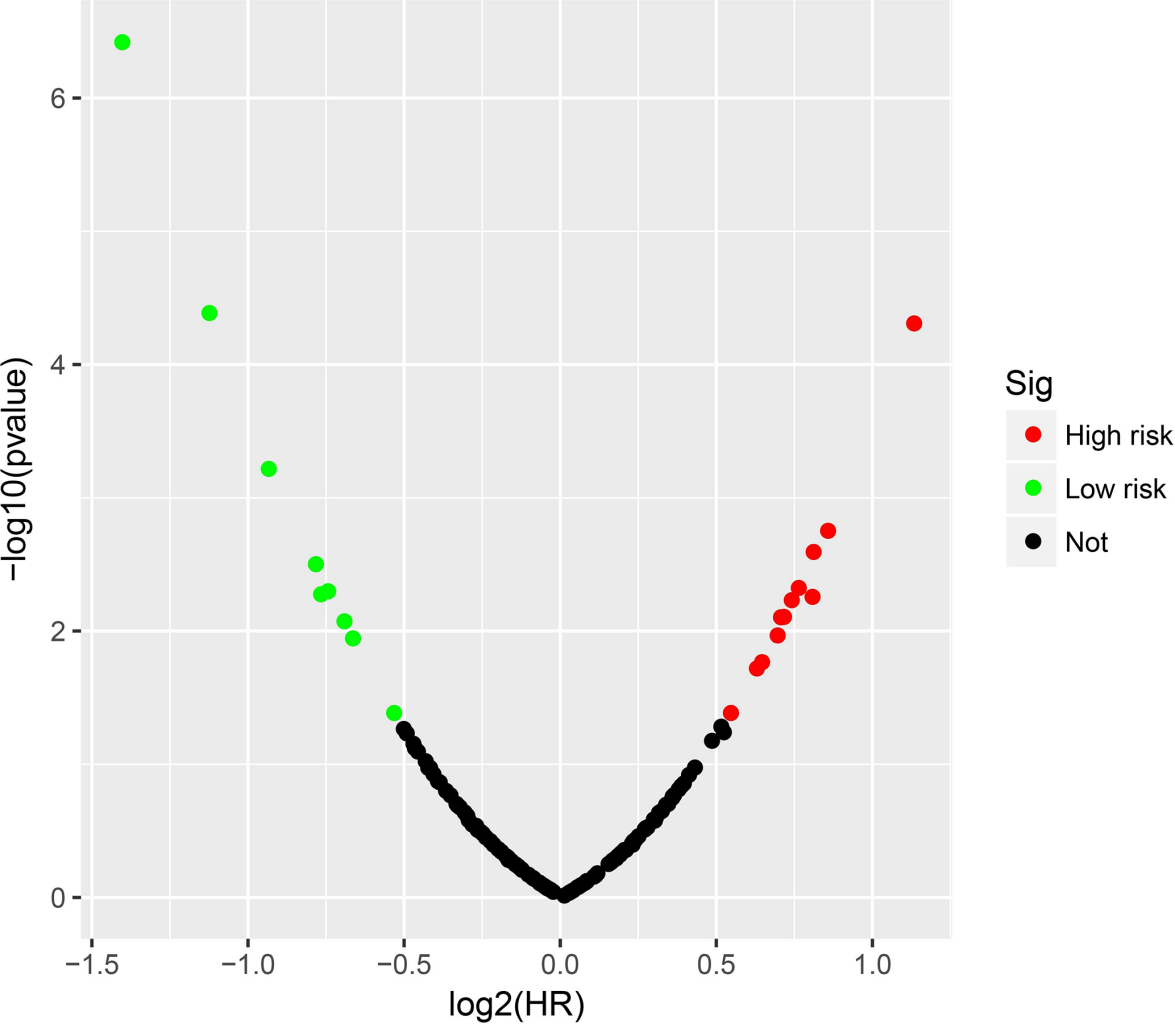
Volcano plot of snoRNAs survival analysis results in TCGA LGG cohort.

**Figure 2:**
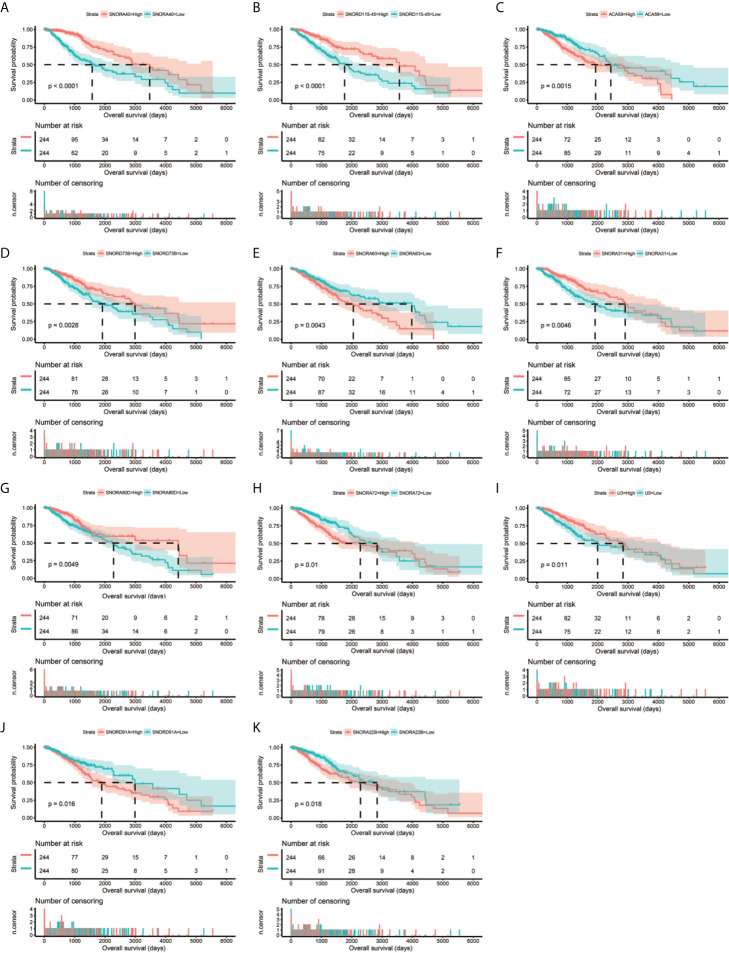
Kaplan–Meier survival curves of 11 snoRNAs that constitute the prognostic signature. SNORA40 **(A)**, SNORD115-45 **(B)**, ACA59 **(C)**, SNORD73B **(D)**, SNORA63 **(E)**, SNORA31 **(F)**, SNORA80D **(G)**, SNORA72 **(H)**, U3 **(I)**, SNORD91A **(J)**, ANORA22B **(K)**.

**Figure 3:**
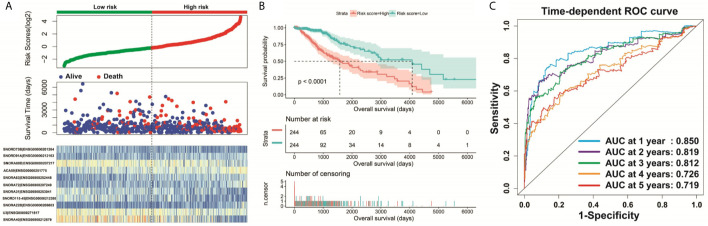
Survival analysis of risk score. **(A)** Distribution of risk score and survival time of LGG patients; **(B)** Kaplan–Meier survival curves of risk score; **(C)** Time-dependent ROC curve of risk score.

**Figure 4:**
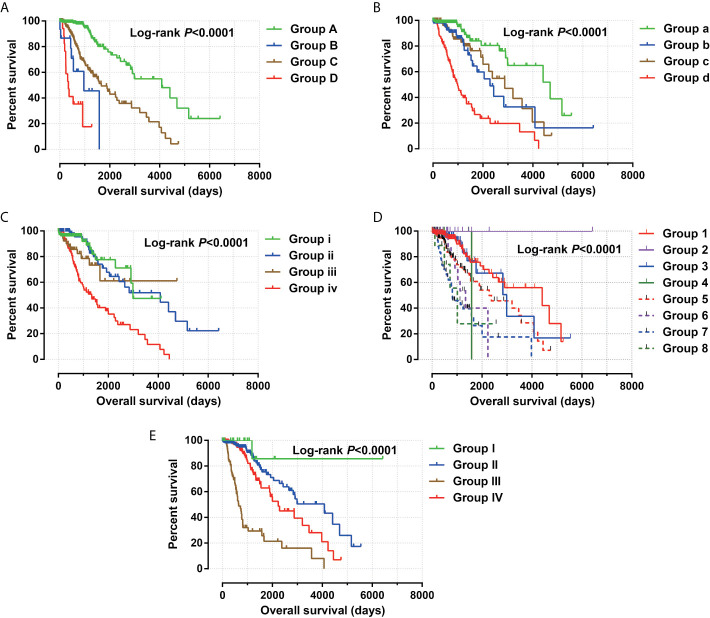
Joint effect survival analysis between risk score and clinical parameters. **(A)** Combination of risk score and age; **(B)** Combination of risk score and grade; **(C)** Combination of risk score and radiation therapy; **(D)** Combination of risk score and tumor location. **(E)** Combination of risk score and IDH1 mutation.

**Figure 5:**
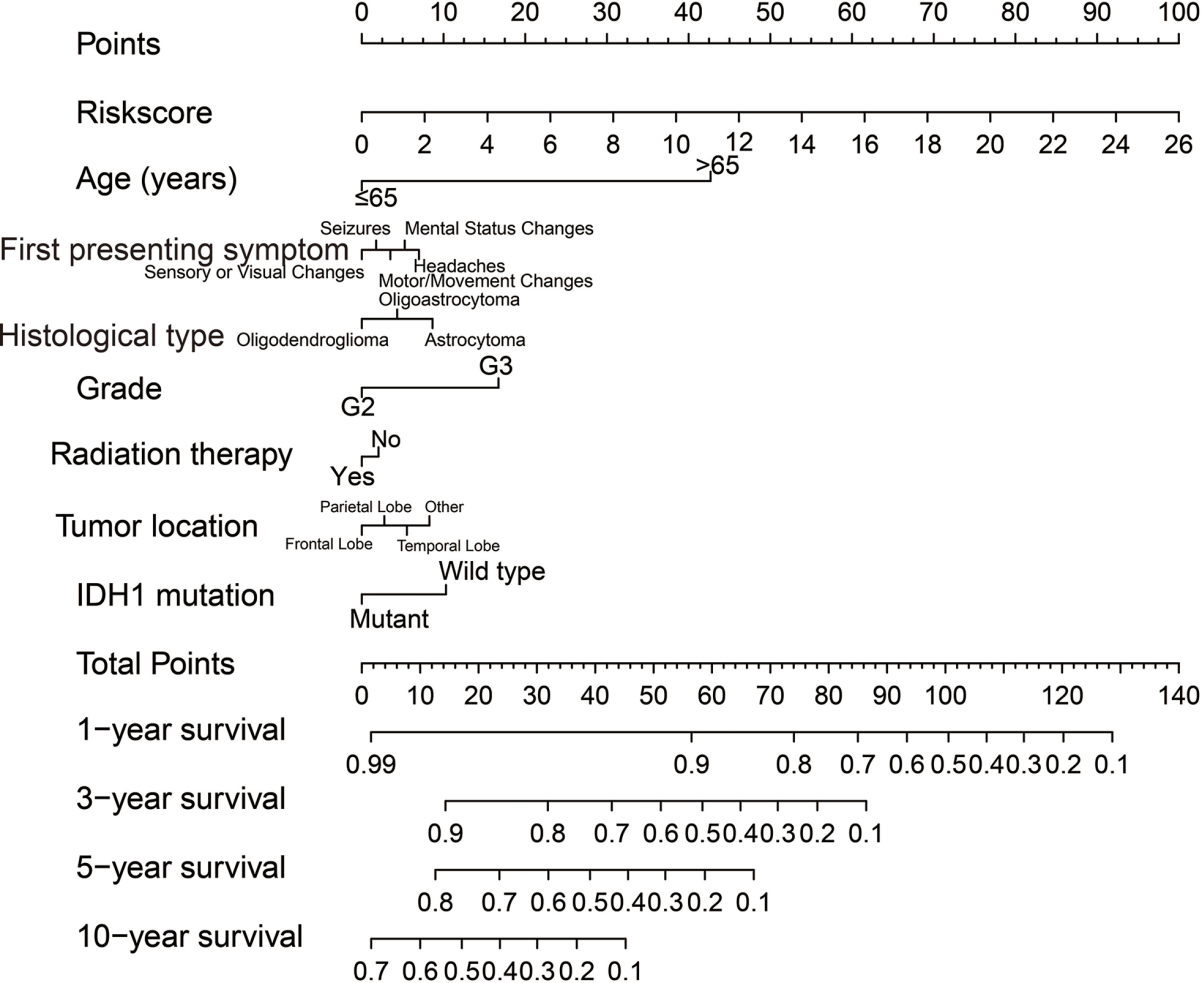
A nomogram constructed in TCGA LGG cohort based on risk score and clinical parameters.

Clinical parameters of TCGA LGG cohort were summarized in **Table 1**. Through survival analysis, we obtained 21 snoRNAs that are markedly connected with the clinical outcome of LGG (**Figure 1** and **Table S1**). Then we use the step function to screen a risk score signature from these 21 prognostic-related snoRNAs. Through the screening of the step function, we finally constructed a signature composed of 11 prognostic snoRNAs. The expression of risk score is below: risk score = (–0.2258) × Exp of SNORD73B + (0.1889) × Exp of SNORD91A + (–0.2338) × Exp of SNORA80D + (0.2783) × Exp of ACA59 + (0.3153) × Exp of SNORA63 + (0.2547) × Exp of SNORA72 + (–0.1293) × Exp of SNORA31 + (–0.2354) × Exp of SNORD115-45 + (0.2076) × Exp of SNORA22B + (–0.2054) × Exp of U3 + (–0.2230) × Exp of SNORA40. Kaplan–Meier curves of 11 prognostic snoRNAs were display in **Figures 2A–K**. By comparing the distribution between survival time of LGG patients and risk score, we found that patients with high risk score had poorer clinical outcome than those with low risk score (**Figures 3A, B**, log rank P <0.0001). We used multivariate Cox proportional hazard regression model to analyze risk score and found that LGG patients with high risk score had a higher risk of death (adjusted P = 0.003, adjusted HR = 2.076, 95%CI = 1.290–3.340). Then we used *survivalROC* to assess the accuracy of this risk score for predicting the clinical outcome of LGG patients. We found that this risk score had the highest accuracy in assess the one year prognosis of LGG patients, and the area under curve (AUC) was 0.850 (**Figure 3C**). The accuracy of the prognosis signature exceeded 0.7 in the clinical outcome prediction of LGG patients from one to five years (**Figure 3C**). We also use this prognostic signature and clinical parameters with P <0.01 to perform a joint effect survival analysis, these clinical parameters including age, grade, IDH1 mutation, radiation therapy and tumor location. The joint effect survival analysis can significantly more accurately classify LGG patients and identify subtypes of LGG patients with different prognostic outcomes (**Table 2** and **Figures 4A–E**). Through the combined effect survival analysis, we found that the combination of age and risk score can significantly divide LGG patients into four subtypes with significant different prognosis (**Table 2** and **Figure 4A**, all adjusted *P <*0.05). For understand the contribution of this prognostic signature to the clinical outcome of LGG patients, we constructed a nomogram base on eight LGG prognostic-related clinical parameters and risk score. Through the nomogram, we found that the risk score has the highest contribution to LGG survival, with scores ranging from 0 to 100 (**Figure 5**).

**Table 1:** Univariate survival analysis of clinical parameter in TCGA LGG cohort.

**Variables**	**Patients**	**MST (days)**	**HR (95% CI)**	**Log-rank *P***
**Race**				0.576
White	451	2,660	1	
Non-white	27	1,578	1.244 (0.578–2.675)	
Missing	10			
**Gender**				0.412
Male	269	2,433	1	
Female	219	2,660	0.861 (0.601–1.232)	
**Age (years)**				<0.001
≤65	454	2,835	1	
>65	34	547	5.877 (3.529–9.786)	
**First presenting symptom**				0.010
Headaches	100	2,379	1	
Mental Status Changes	37	1,351	2.124 (1.101–4.097)	
Motor/Movement Changes	36	2,286	1.857 (0.909–3.792)	
Seizures	238	2,835	0.968 (0.606–1.546)	
Sensory or Visual Changes	28	4,695	0.532 (0.203–1.393)	
Missing	49			
**First presenting symptom longest duration**				0.075
0–30 Days	207	2,052	1	
31–90 Days	75	2,379	1.07 (0.644–1.779)	
91–180 Days	35	1,491	1.314 (0.702–2.46)	
>181 Days	105	2,988	0.591 (0.361–0.969)	
Missing	66			
**Histological_type**				0.011
Astrocytoma	184	1,891	1	
Oligoastrocytoma	122	3,200	0.628 (0.392–1.006)	
Oligodendroglioma	182	2,907	0.559 (0.373–0.839)	
**Laterality**				0.183
Left	236	1,762	1	
Midline	6	682	0.947 (0.283–3.172)	
Right	241	2,907	0.713 (0.494–1.027)	
Missing	5			
**Grade**				<0.001
G2	237	3,571	1	
G3	250	1,525	3.431 (2.316–5.083)	
Missing	1			
**Preoperative antiseizure meds**				0.257
No	104	2,988	1	
Yes	253	2,660	1.335 (0.809–2.204)	
Missing	131			
**Preoperative corticosteroids**				0.052
No	203	2,988	1	
Yes	145	2,433	1.553 (0.994–2.427)	
Missing	140			
**Radiation therapy**				0.002
No	168	2,988	1	
Yes	276	2,000	2.033 (1.294–3.193)	
Missing	44			
**Targeted molecular therapy**				0.115
No	186	2,907	1	
Yes	251	2,282	1.369 (0.925–2.026)	
Missing	51			
**Tumor location**				0.002
Frontal Lobe	288	3,200	1	
Parietal Lobe	44	2,235	1.042 (0.498–2.182)	
Temporal Lobe	137	1,666	2.009 (1.369–2.949)	
Other	18	1,585	2.318 (0.928–5.790)	
Missing	1			
**IDH1 mutation**				<0.001
Wild type	117	775	1	
Mutant	371	2,907	0.264 (0.184–0.379)	

TCGA, The Cancer Genome Atla; LGG, lower grade glioma; MST, median survival time; HR, hazard ratio; CI, confidence interval; NA, not available.

**Table 2:** Joint effect survival analysis of the prognostic signature and clinical parameters with *P <*0.01.

**Group**	**Risk score**	**Variables**	**Patients (n = 488)**	**MST (days)**	**Crude HR (95% CI)**	**Crude *P***	**Adjusted HR (95% CI)**	**Adjusted *P*§**
		**Age (years)**						
**A**	**Low risk**	**≤65**	228	4,084	1		1	
**B**	**Low risk**	**>65**	16	962	8.270 (3.568–19.164)	<0.001	9.172 (2.912–28.888)	<0.001
**C**	**High risk**	**≤65**	226	1,666	3.552 (2.305–5.474)	<0.001	2.328 (1.411–3.841)	0.001
**D**	**High risk**	**>65**	18	347	18.841 (9.335–38.029)	<0.001	6.477 (2.776–15.113)	<0.001
		**Grade£**						
**a**	**Low risk**	**G2**	138	4,695	1		1	
**b**	**Low risk**	**G3**	105	2,282	2.432 (1.263–4.684)	0.008	1.884 (0.897–3.956)	0.094
**c**	**High risk**	**G2**	99	2,875	2.278 (1.182–4.389)	0.014	1.740 (0.826–3.668)	0.145
**d**	**High risk**	**G3**	145	987	8.259 (4.687–14.552)	<0.001	4.346 (2.180–8.662)	<0.001
		**IDH1 mutation**						
**I**	**Low risk**	**Wild type**	20	NA	1		1	
**II**	**Low risk**	**Mutant**	224	4,084	3.735 (0.509–27.387)	0.195	5.158 (0.601–44.300)	0.135
**III**	**High risk**	**Wild type**	97	648	26.906 (3.666–197.488)	0.001	26.208 (3.027–226.875)	0.003
**IV**	**High risk**	**Mutant**	147	2,235	6.348 (0.861–46.789)	0.070	7.157 (0.833–61.474)	0.073
		**Radiation therapy¥**						
**i**	**Low risk**	**No**	107	2,988	1		1	
**ii**	**Low risk**	**Yes**	121	4,084	1.084 (0.541–2.172)	0.821	0.523 (0.240–1.140)	0.103
**iii**	**High risk**	**No**	61	NA	1.753 (0.783–3.926)	0.173	1.014 (0.430–2.394)	0.974
**iv**	**High risk**	**Yes**	155	1,335	4.187 (2.312–7.584)	<0.001	1.440 (0.711–2.919)	0.311
		**Tumor location** ɠ						
**1**	**Low risk**	**Frontal Lobe**	161	NA	1		1	
**2**	**Low risk**	**Parietal Lobe**	18	NA	7 × 10^–6^ (2.218 × 10^−184^–2.460 × 10^173^)	0.955	8.792 × 10^−7^ (6.370 × 10^−239^–1.214 × 10^226^)	0.959
**3**	**Low risk**	**Temporal Lobe**	59	NA	1.095 (0.527–2.274)	0.808	1.103 (0.519–2.347)	0.799
**4**	**Low risk**	**Other**	6	NA	1.828 (0.246–13.560)	0.555	NA	NA
**5**	**High risk**	**Frontal Lobe**	127	NA	2.145 (1.294–3.554)	0.003	1.256 (0.701–2.249)	0.444
**6**	**High risk**	**Parietal Lobe**	26	NA	3.466 (1.547–7.766)	0.003	2.083 (0.856–5.067)	0.106
**7**	**High risk**	**Temporal Lobe**	78	NA	5.787 (3.439–9.736)	<0.001	2.655 (1.325–5.321)	0.006
**8**	**High risk**	**Other**	12	NA	4.738 (1.640–13.690)	0.004	2.374 (0.653–8.635)	0.189

£ Grade information is unavailable in one patient; ¥ Radiation therapy information are unavailable in 44 patients; ɠ Tumor location information are unavailable in one patients. §adjusted for age, first presenting symptom, histological type, grade, radiation therapy, tumor location, IDH1 mutation.

MST, median survival time; HR, hazard ratio; CI, confidence interval; NA, not available.

### Functional Enrichment Analysis of snoRNAs Signature

**Figure 6:**
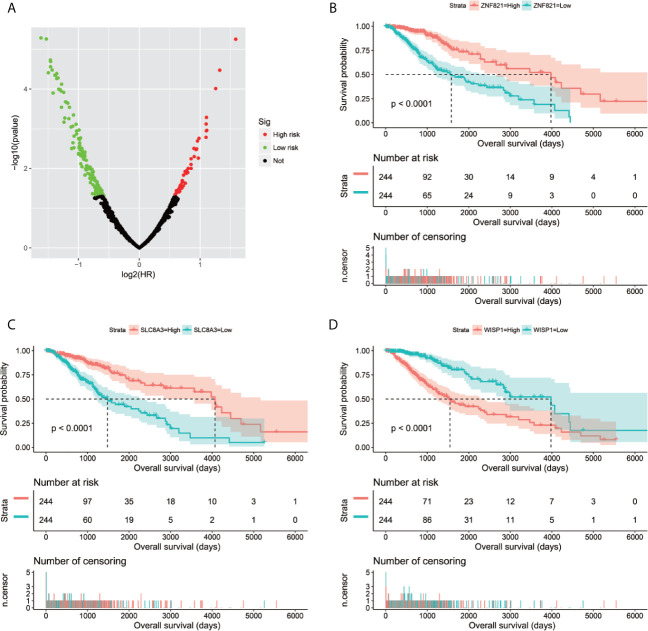
Survival analysis results of snoRNAs co-expressed PCGs in TCGA LGG cohort. **(A)** Volcano plot of snoRNAs co-expressed PCGs survival analysis results; **(B)** Kaplan–Meier survival curves of ZNF821; **(C)** Kaplan–Meier survival curves of SLC8A3; **(D)** Kaplan–Meier survival curves of WISP1.

Through genome-wide co-expression analysis, 990 co-expression pairs of snoRNAs-PCGs were identified, and the snoRNAs-PCGs co-expression interaction network is shown in **Figure S1**, since we did not screen the co-expressed PCGs of SNORD91A in this cohort. Finally, we will screen the co-expressed genes of 10 snoRNAs into the subsequent functional enrichment analysis. Enrichment analysis reveals that these snoRNA co-expressed PCGs may work by participating in the following biological functions: DNA repair, neuromuscular process controlling balance, covalent chromatin modification, neurotransmitter secretion, voltage-gated calcium channel activity, NIK/NF-kappaB signaling, nervous system development, neuromuscular junction, neuronal action potential, protein polyubiquitination, chemical synaptic transmission, brain morphogenesis, regulation of long-term neuronal synaptic plasticity, positive regulation of GTPase activity, MAPK cascade, positive regulation of cyclin-dependent protein serine/threonine kinase activity involved in G1/S transition of mitotic cell cycle, protein kinase binding, regulation of cell cycle, synaptic vesicle cycle and glutamatergic synapse (**Table S3**). We also performed a multivariate survival analysis on these snoRNA co-expressed PCGs, and obtained 180 prognostic PCGs of LGG (**Figure 6A**). The top three significant PCGs are zinc finger protein 821 (ZNF821, **Figure 6B**), solute carrier family 8 member A3 (SLC8A3, **Figure 6C**) and cellular communication network factor 4 (CCN4, also named as WISP1, **Figure 6D**).

**Figure 7:**
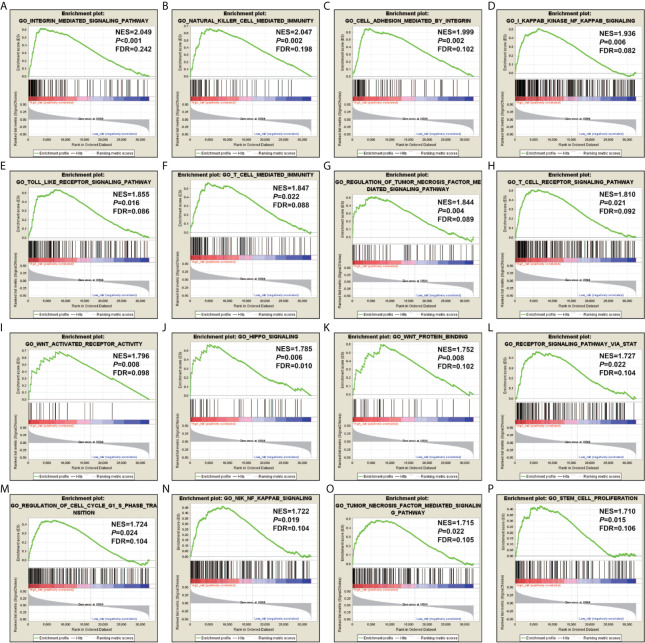
GSEA results using the c5 reference gene set. **(A)** integrin mediated signaling pathway; **(B)** natural killer cell mediated immunity; **(C)** cell adhesion mediated by integrin; **(D)** kappa B kinase NF kappa B signaling; **(E)** toll like receptor signaling pathway; **(F)** T cell mediated immunity; **(G)** regulation of tumor necrosis factor mediated signaling pathway; **(H)** T cell receptor signaling pathway; **(I)** Wnt activated receptor activity; **(J)** hippo signaling; **(K)** Wnt protein binding; **(L)** receptor signaling pathway *via* STAT; **(M)** regulation of cell cycle G1/S phase transition; **(N)** NIK/NF kappa B signaling; **(O)** tumor necrosis factor mediated signaling pathway; **(P)** stem cell proliferation.

In order to reveal the molecular mechanisms that cause the prognosis difference of LGG patients with high- and low-risk score phenotypes, we also compared patients with two phenotypes using the GSEA approach. GSEA using the c5 reference gene set suggested that LGG patients with high-risk score phenotypes are significantly different from low-risk score phenotypes in the following biological processes: integrin mediated signaling pathway, natural killer cell mediated immunity, cell adhesion mediated by integrin, kappa B kinase NF kappa B signaling, toll like receptor signaling pathway, T cell mediated immunity, regulation of tumor necrosis factor mediated signaling pathway, T cell receptor signaling pathway, Wnt activated receptor activity, hippo signaling, Wnt protein binding, receptor signaling pathway *via* STAT, regulation of cell cycle G1/S phase transition, NIK/NF kappa B signaling, tumor necrosis factor mediated signaling pathway, stem cell proliferation (**Figures 7A–P**). GSEA using the c2 reference gene set suggested that LGG patients with high-risk score phenotypes are significantly different from low-risk score phenotypes in the following pathways: CD8/TCR pathway, natural killer cell mediated cytotoxicity, B cell receptor signaling pathway, PI3KCI pathway, caspase pathway, TH1/TH2 pathway, P53 pathway, metastasis up, tumor vasculature up, JAK/STAT signaling pathway, cell adhesion molecules CAMs, P38/MAPK pathway, Wnt pathway require Myc, signaling by Rho gtpases, tumorigenesis up and NF-κB pathway (**Figures S2A–P**).

**Figure 8:**
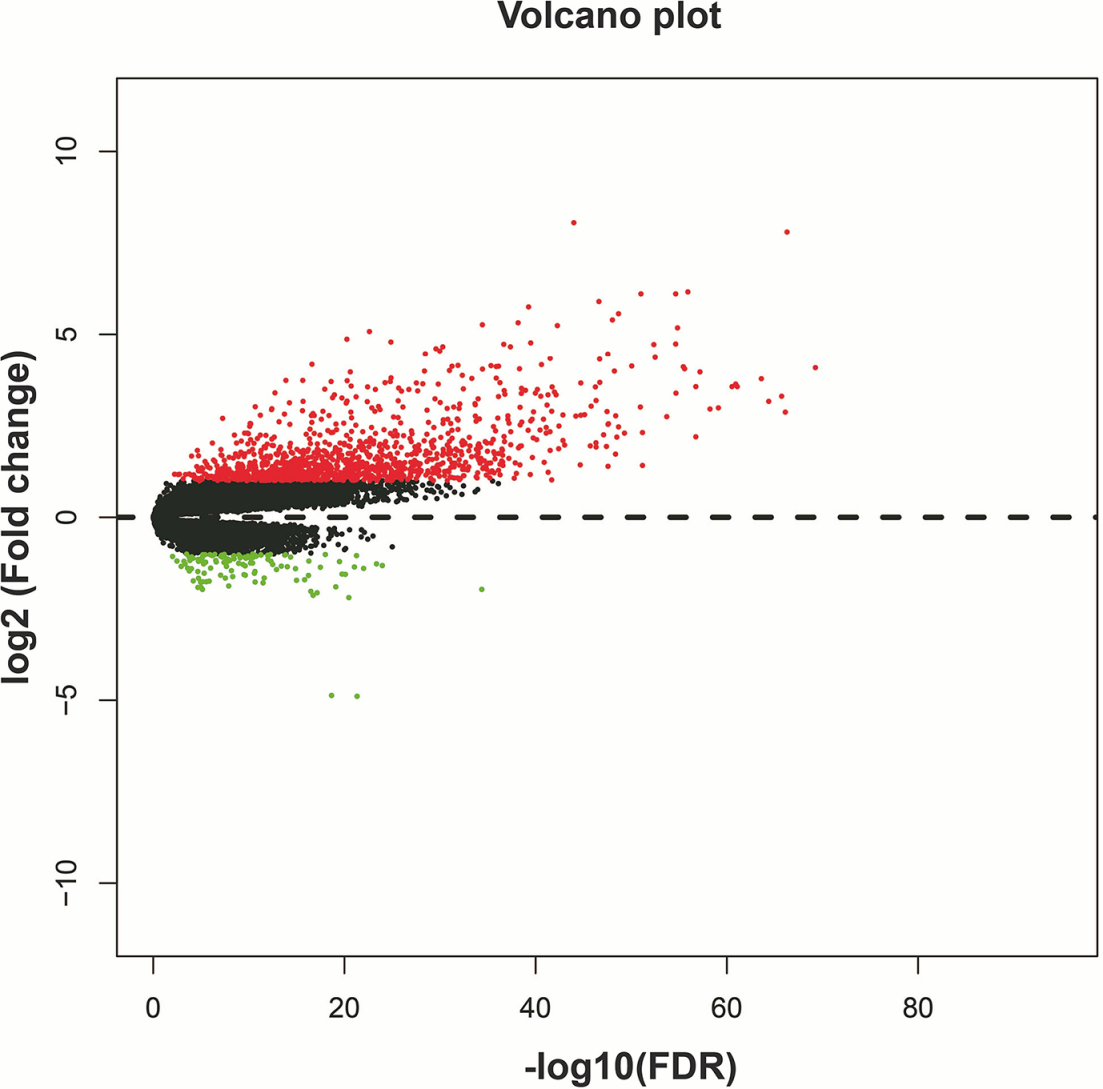
Volcano plot of DEGs between high- and low-risk score phenotypes.

**Figure 9:**
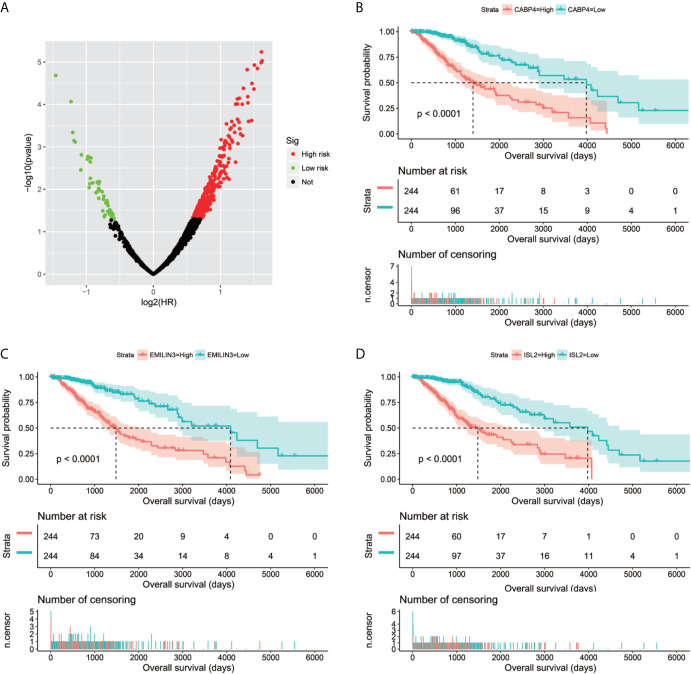
Survival analysis results of DEGs between high- and low-risk score phenotypes in TCGA LGG cohort. **(A)** Volcano plot of DEGs survival analysis results; **(B)** Kaplan–Meier survival curves of CABP4; **(C)** Kaplan–Meier survival curves of EMILIN3; **(D)** Kaplan–Meier survival curves of ISL2.

**Figure 10:**
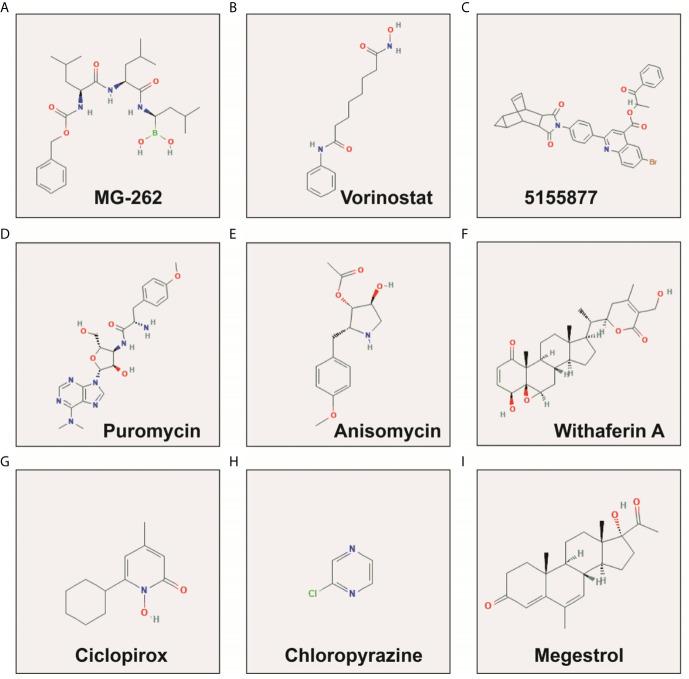
The chemical structure of the targeted therapeutic drugs of snoRNAs prognostic signature in LGG. **(A)** MG-262; **(B)** Vorinostat; **(C)** 5155877; **(D)** Puromycin; **(E)** Anisomycin; **(F)** Withaferin A; **(G)** Ciclopirox; **(H)** Chloropyrazine; **(I)** Megestrol.

We screened 1319 DEGs between high- and low-risk score phenotypes by *edgeR*, of them, 122 DEGs were down-regulation and 1197 DEGs were up-regulation (**Figure 8**, **Figure S3** and **Table S7**). Functional enrichment revealed that these DEGs may function by participating in the following biological processes: positive regulation of cell proliferation, cell-cell signaling, positive regulation of ERK1 and ERK2 cascade, interferon-gamma-mediated signaling pathway, regulation of immune response, neuron migration, angiogenesis, positive regulation of cell migration, cytokine-mediated signaling pathway, cell adhesion, cellular response to interleukin-1, cell differentiation, positive regulation of T cell proliferation, positive regulation of cytosolic calcium ion concentration involved in phospholipase C-activating G-protein coupled signaling pathway, cell surface receptor signaling pathway, neuropeptide signaling pathway, JAK-STAT cascade, mitotic sister chromatid segregation, cell division, mitotic nuclear division, integrin binding, cell-matrix adhesion, T cell receptor signaling pathway, response to hypoxia, positive regulation of protein kinase B signaling, epithelial to mesenchymal transition, regulation of vascular endothelial growth factor production, positive regulation of MAPK cascade, cellular response to fibroblast growth factor stimulus, phosphatidylinositol-4,5-bisphosphate 3-kinase activity, Wnt-protein binding, cytokine–cytokine receptor interaction, ECM-receptor interaction, cell adhesion molecules (CAMs), focal adhesion, PI3K-Akt signaling pathway, Proteoglycans in cancer, cell cycle, chemokine signaling pathway, Jak-STAT signaling pathway, and transcriptional misregulation in cancer (**Table S8**). We also performed a multivariate survival analysis on these DEGs, and obtained 320 prognostic DEGs of LGG (**Table S9** and **Figure 9A**). The top three significant DEGs were calcium binding protein 4 (CABP4, **Figure 9B**), elastin microfibril interfacer 3 (EMILIN3, **Figure 9C**) and ISL LIM homeobox 2 (ISL2, **Figure 9D**). We use these DEGs through the CMAP to identify the potential targeted therapy drugs of this snoRNAs prognostic signature in LGG. We have obtained ten targeted therapy drugs for this snoRNAs prognostic signature in LGG. These drugs are 15-delta prostaglandin J2, MG-262, vorinostat, 5155877, puromycin, anisomycin, withaferin A, ciclopirox, chloropyrazine and megestrol (**Table 3**). The chemical structures of nine drugs (except 15-delta prostaglandin J2) can be obtained through PubChem, which are shown in **Figures 10A–I**. Subsequently, we also constructed the drug-genes interaction network by STITCH, In addition to 15-delta prostaglandin J2 and 5155877, we can obtain drug-genes interaction network for the remaining 8 drugs (**Figure S4**). We found that these drugs can work by regulating genes that are differentially expressed between high and low-risk score phenotypes. MG-262 may function in LGG by regulating cyclin dependent kinase 4 (CDK4), NME/NM23 family member 8 (NME8), chloride intracellular channel 6 (CLIC6) and acyl-coa dehydrogenase long chain (ACADL), while withaferin A through regulating tubulin beta 6 class V (TUBB6), vimentin (VIM), DLG associated protein 5 (DLGAP5), CDK4, tubulin alpha 1c (TUBA1C), kinesin family member C1 (KIFC1). Vorinostat was function by regulating UDP glucuronosyltransferase family 2 member B17 (UGT2B17), suppressor of cytokine signaling 3 (SOCS3), epidermal growth factor receptor (EGFR), Fas ligand (FASLG) and matrix metallopeptidase 9 (MMP9). Ciclopirox was function by regulating lactotransferrin (LTF), arachidonate 15-lipoxygenase (ALOX15) and baculoviral IAP repeat containing 5 (BIRC5). Megestrol was function by regulating gamma-aminobutyric acid type A receptor subunit gamma 2 (GABRG2) and neuronal PAS domain protein 4 (NPAS4). Anisomycin was function by regulating MMP9, dual specificity phosphatase 9 (DUSP9), EGFR, interleukin 6 (IL6), resistin (RETN), growth differentiation factor 15 (GDF15), C-C motif chemokine ligand 20 (CCL20), proteoglycan 4 (PRG4) and mitogen-activated protein kinase 15 (MAPK15). Puromycin was function by regulating actin gamma 2, smooth muscle (ACTG2), fibronectin 1 (FN1), ATP binding cassette subfamily C member 3 (ABCC3), dipeptidyl peptidase 4 (DPP4), EGFR and syndecan 1 (SDC1).

**Table 3:** Connectivity map analysis results.

**CMap name**	**Mean connective score**	**n**	**Enrichment**	***P* value**	**Specificity**	**Percent non-null**
**15-delta prostaglandin J2**	–0.393	15	–0.584	<0.001	0.0526	53
**MG-262**	–0.6	3	–0.837	0.00865	0.126	66
**Vorinostat**	–0.412	12	–0.45	0.0092	0.4867	50
**5155877**	–0.408	4	–0.725	0.01172	0.0197	50
**Puromycin**	–0.614	4	–0.722	0.01215	0.097	75
**Anisomycin**	–0.366	4	–0.71	0.01456	0.0932	50
**Withaferin A**	–0.542	4	–0.691	0.01959	0.1393	75
**Ciclopirox**	–0.385	4	–0.682	0.02244	0.0762	50
**Chloropyrazine**	–0.37	4	–0.671	0.02604	0.039	50
**Megestrol**	–0.393	4	–0.635	0.04323	0.034	50

### Tumor Immune Infiltration and Microenvironment Analysis

**Figure 11:**
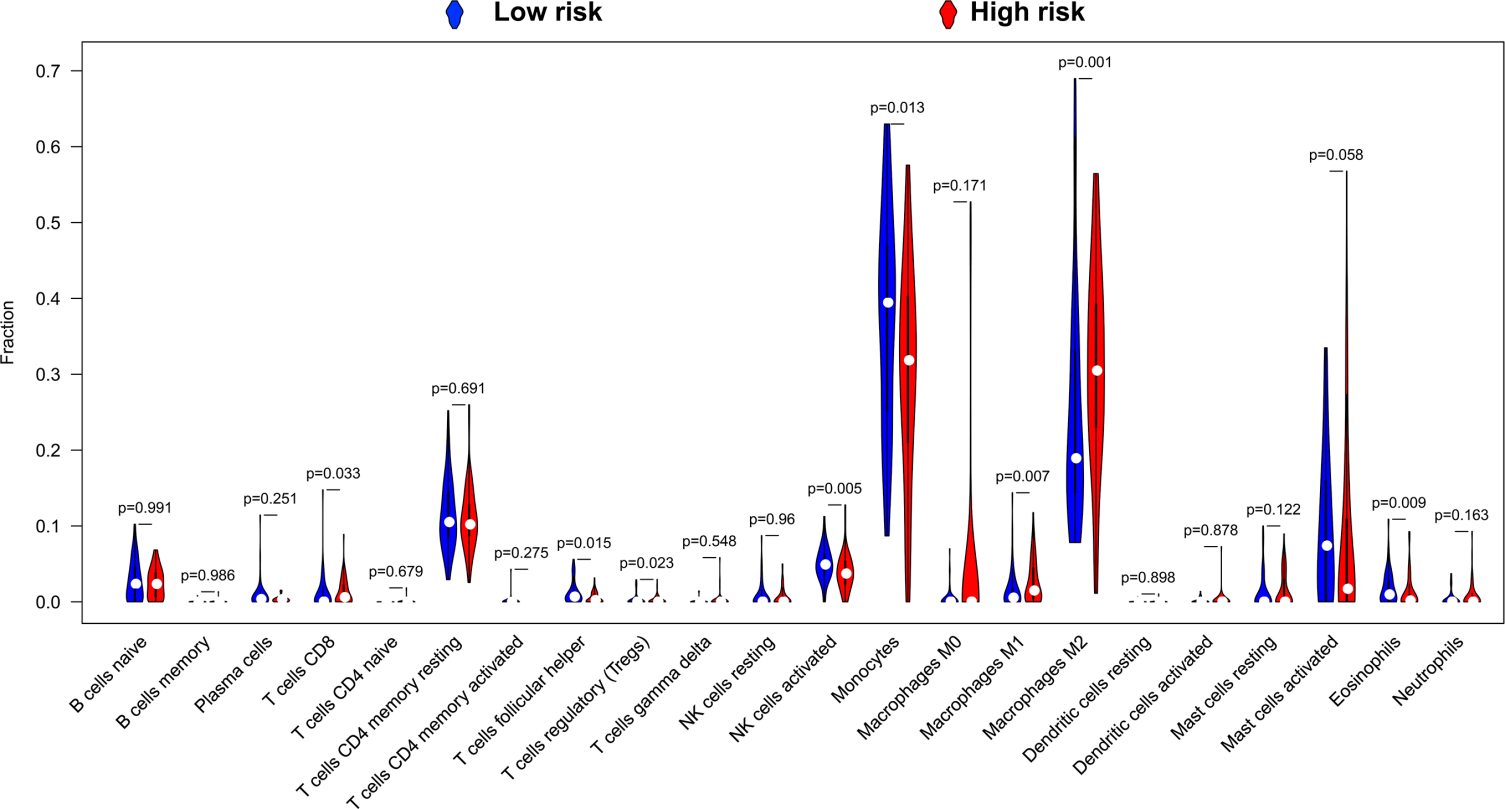
Violin plot of 22 immune cells infiltration between high- and low-risk score phenotypes.

**Figure 12:**
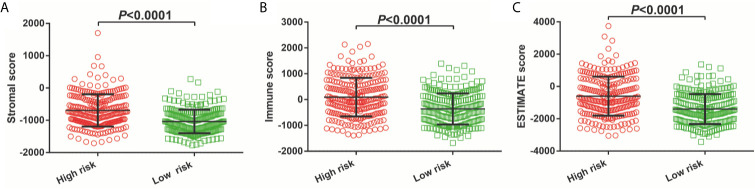
Immune microenvironment score between high- and low-risk score phenotypes. **(A)** Stromal score; **(B)** Immune score; **(C)** ESTIMATE score.

By using CIBERSORT to assess the degree of infiltration of 22 immune cells in LGG tumor tissues, we finally obtained distribution histogram and abundance heatmap of immune cell infiltration of 116 LGG patients (**Figures S5A–B**), of which 52 patients were the low-risk score phenotype and 64 patients were high-risk score phenotype. By comparing the distribution of 22 immune cells between high- and low-risk score phenotypes of LGG patients, we found that eight immune cells, including T cell CD8, T cells follicular helper, T cells regulatory (Tregs), NK cells activated, Monocytes, Macrophages M1, Macrophages M2 and Eosinophils, showed significant differences between high- and low-risk score phenotypes (**Figure 11**). Among the eight types of immune cells, four types of immune cells [T cell CD8, T cells regulatory (Tregs), Macrophages M1and Macrophages M2] in the high-risk phenotype have a higher fraction than in the low-risk phenotype, whereas, four types of immune cells (T cells follicular helper, NK cells activated, Monocytes and Eosinophils) in the low-risk phenotype have a higher fraction than these in the high-risk phenotype (**Figure 11**). Subsequently, we also scored the immune cells and stromal cells in tumor tissues of 488 LGG patients. We found that all the stromal, immune, and ESTIMATE score for the tumor microenvironment of LGG patients were higher in the high-risk score phenotype (**Figures 12A–C**).

## Discussion

There has been a small amount of literature that used the TCGA genome-wide data set to screen snoRNA prognostic biomarkers. Liu et al. used TCGA genome-wide RNA-seq dataset to investigate sarcoma prognostic-related snoRNAs, and identified 15 sarcoma prognostic-related snoRNAs, as well as constructed a sarcoma prognostic signature base on four snoRNAs (24). Cao et al. carried out screening for prognostic snoRNAs by using the least absolute and selection operator (LASSO) Cox regression method, and developed a bladder cancer prognostic signature composed of five snoRNAs (25). Both of the above studies confirmed that U3 can be used as a prognostic marker for corresponding tumors (24, 25). Similarly, Zhao et al. also constructed a renal clear cell carcinoma with six prognosis-related snoRNA signatures, and conducted functional enrichment analysis on these snoRNAs (26). Similar to previous studies, our current study also used TCGA RNA-seq dataset to investigate prognostic snoRNAs, and also found that U3 can be used as a prognostic biomarker for LGG. Therefore, we inferred that U3 could be used as prognostic biomarker in multiple cancers.

Lafaille et al. found that the genetic variation of SNORA31 was closely related to encephalitis in the forebrain. After alleles at corresponding sites of SNORA31 were knocked out, *in vitro* experiments proved that SNORA31 could change the sensitivity of neurons to herpes simplex virus-1 (27). The expression of SNORA31 was markedly down-regulated in CD19+ b-cells of chronic myeloid leukemia (CLL) patients compared with normal B-cells (28). Studies have shown that relapsing–remitting multiple sclerosis (RRMS) patients ncRNA–mRNA network is seriously affected by the disease, resulting in a large number of ncRNAs and mRNAs imbalance. By means of RNA sequencing, Irizar et al. found that SNORA40 was disorder in RRMS and might be a potential therapeutic target (29). We found that the rest of snoRNAs had not been reported before. We found for the first time that that these snoRNAs are related to LGG prognosis through whole-genome RNA sequencing data. Through the snoRNAs co-expression genes, GSEA and differentially expressed genes, we have a further understanding the function of this eleven-snoRNA prognostic signature. A large number of pathways and biological function gene sets enriched by this eleven-snoRNA prognostic signature have been reported as classic or novel cancer-related signaling pathways in previous studies, such as JAK/STAT, p38//MAPK, and Wnt signaling pathways.

Xiong et al. screened the differentially expressed genes between ovarian serous cystadencinoma (OSC) and ovarian specimens, and used CMap method to determine that three drugs, namely MG-132, puromycin and 15-delta prostaglandin J2, could be potential target drugs for OSC (30). Shi et al. also identified 15-delta Prostaglandin J2 as a potential therapeutic agent by comparing gene expression profiles in patients with diabetic nephropathy using bioinformatics analysis (31). Wei et al. through genome-wide data and CMAP analysis identified MC-262 as a potential targeted therapy for head and neck squamous cell carcinoma (HNSCC), and its potential target in HNSCC is proliferating cell nuclear antigen (32). Vorinostat in the treatment of glioma has been widely reported in previous studies. Clinical trials have shown that vorinostat combined with Bevacizumab or Temozolomide can be used in the treatment of glioma (33–35), as well recurrent malignant gliomas (36–39). Local Delivery of vorinostat can alter the tumor microenvironment, thereby inhibiting glioma recurrence (40). Vorinostat can also mediate the regulation of cell cycle regulatory proteins in glioma (41). Bortezomib can enhance the apoptosis of glioma cells induced by vorinostat (42). Puromycin exerts antitumor function in multiple cancers and can induce cancer cell apoptosis. The aminopeptidase inhibitors based on puromycin show high anti-tumor effect *in vitro* in hematologic malignancies and are expected to be potential therapeutic drugs for hematologic diseases (43). Puromycin can induce apoptosis of breast cancer cells MCF-7. Its apoptosis mechanism is not exerted by insulin-like growth factor-1 (IGF-1), but is affected by the level of IGF-1 receptor (44). Study have shown that puromycin can induce apoptosis of glioma cells, but its apoptotic mechanism is not exerted through the Fas/Fas ligand pathway and Bcl-2 has only a small protective effect on puromycin-induced apoptosis of glioma cells (45). Previous studies have shown that anisomycin has anticancer effects in a variety of cancers, but its molecular mechanisms are different in different cancers. Anisomycin can inhibit angiogenesis in ovarian cancer (OV) through take part in the ceRNA regulatory network (lncRNA−Meg3/miR−421/platelet derived growth factor receptor α axis), thereby inhibiting tumor cell growth (46). LncRNA β-site APP cleaving enzyme 1 antisense strand (BACE1-AS) is also a potential target of anisomycin in OV (47). Anisomycin plays an anti-tumor role in primary hepatocellular carcinoma by mediating natural killer cells, and its main molecular mechanism has been found by genome sequencing to be mediated by immunomodulatory genes (48). Anisomycin can be involved in inhibiting the proliferation of colorectal cancer cells by mediating GATA binding protein 6 (49). Anisomycin can improve the efficacy of BCR-ABL tyrosine kinase inhibitors in CLL by mediating Wnt/β-catenin signaling pathway (50). Anisomycin can also mediate phosphorylated mitogen-activated protein kinases (MAPKs) p38 and Jun N-terminal kinase (JNK) to increase the apoptosis of glucocorticoids-resistant acute lymphoblastic leukemia cell lines (51). Anisomycin can also increase the sensitivity of melanoma anti-tumor drugs (52). Anisomycin exerting an anti-tumor effect in osteosarcoma through induces cell cycle arrest and apoptosis by mediating mitochondrial biogenesis (53). Anisomycin in renal cell carcinoma cells can mediate Bcl-2/c-FLIP(L)/Mcl-1/death receptor 4 (DR4) to promote cell apoptosis and exert anti-tumor effects (54, 55). Anisomycin induces apoptosis of glioma cell lines (U251 and U87 cell lines) by down-regulating PP2A catalytic subunit in *in vitro* cell experiments (56). Studies found that withaferin A has great potential in anti-tumor therapy (57, 58), as well as in brain tumor (59, 60). The combination of Withaferin A and tumor treating fields can improve its anti-tumor effect (61). The potential molecular mechanism of Withaferin A in glioma may be exerted by NF-KB nuclear translocation, activation of caspase cascade and activating transcription factor 4(ATF4)-ATF3-CHOP axis (62, 63). Grogan et al. found that withaferin A can exert anti-tumor effects in glioblastoma multiforme through the Akt/mTOR signaling pathway, block the cell cycle of glioblastoma multiforme cells, induce apoptosis, and inhibit cell proliferation and invasion (64, 65). Ciclopirox is also a widely reported anti-tumor drug (66–69). However, after reviewing the literature, we have not found an anti-tumor study of ciclopirox in glioma. Megestrol exert anti-tumor effects in multiple cancers, but has not been reported in the treatment of glioma (70–72). Among the ten drugs we screened, 5155877 and chloropyrazine have not been reported in anti-tumor studies.

This study still has some limitations that need to be clarified. First, this study is a single-center study and lacks a validation cohort. Therefore, our results need further validation in a multi-center, large sample cohort. Second, the molecular mechanism analysis and drug prediction of snoRNA in this study are based on bioinformatics analysis methods, so our results require further verification by *in vivo* and *in vitro* experiments. Despite the above limitations, our research is still the first study to screen LGG prognosis snoRNAs based on whole-genome RNA-seq dataset. Our research is also the first to conduct a comprehensive analysis of LGG prognosis snoRNAs, including molecular mechanism, targeted drug prediction, tumor immune infiltration and tumor microenvironment. The above results can provide a basis for future study on clinical application and molecular mechanisms of snoRNAs in LGG.

## Conclusion

In conclusion, our current study have identified 21 prognostic snoRNAs and constructed a novel eleven-snoRNA prognostic signature for LGG patients. GSEA and functional enrichment analysis suggest that this signature may be involved in the following biological processes and signaling pathways: such as cell cycle, Wnt, mitogen-activated protein kinase, janus kinase/signal transducer and activator of tran-ions, T cell receptor, nuclear factor-kappa B signaling pathway. CMap analysis screened out ten targeted therapy drugs for this signature: 15-delta prostaglandin J2, MG-262, vorinostat, 5155877, puromycin, anisomycin, withaferin A, ciclopirox, chloropyrazine and megestrol. We also found that high- and low-risk score phenotypes of LGG patients have significant differences in immune infiltration and cancer immune microenvironment. The novel findings of our study are the first comprehensive genome-wide investigation of the prognostic related snoRNAs in LGG and the preliminary identification of a novel prognostic snoRNAs signature. We used the bioinformatics analysis to identify the prognostic value and biological mechanisms of this signature. Since this study is based on a single-center study, our results still need to be further verified.

## Data Availability Statement

The original contributions presented in the study are included in the article/**Supplementary Material**. Further inquiries can be directed to the corresponding author.

## Author Contributions

TD: Study design and preparation of manuscript (equal contributors). YG: Study planning and literature search (equal contributors). XL: Data collection and analysis. XW: Literature analysis. XZ: Literature analysis. GZ: Data interpretation. LM: Study planning. All authors contributed to the article and approved the submitted version.

## Conflict of Interest

The authors declare that the research was conducted in the absence of any commercial or financial relationships that could be construed as a potential conflict of interest.
