# Supplementary material for: Integrative Analysis of a Novel Eleven-Small Nucleolar RNA Prognostic Signature in Patients With Lower Grade Glioma
Source: Front Oncol. 2021 Jun 7;11:650828. doi: 10.3389/fonc.2021.650828 (PMC8215672; doi:10.3389/fonc.2021.650828)
Supplement: Supplementary file 2 [file DataSheet_2.pdf]

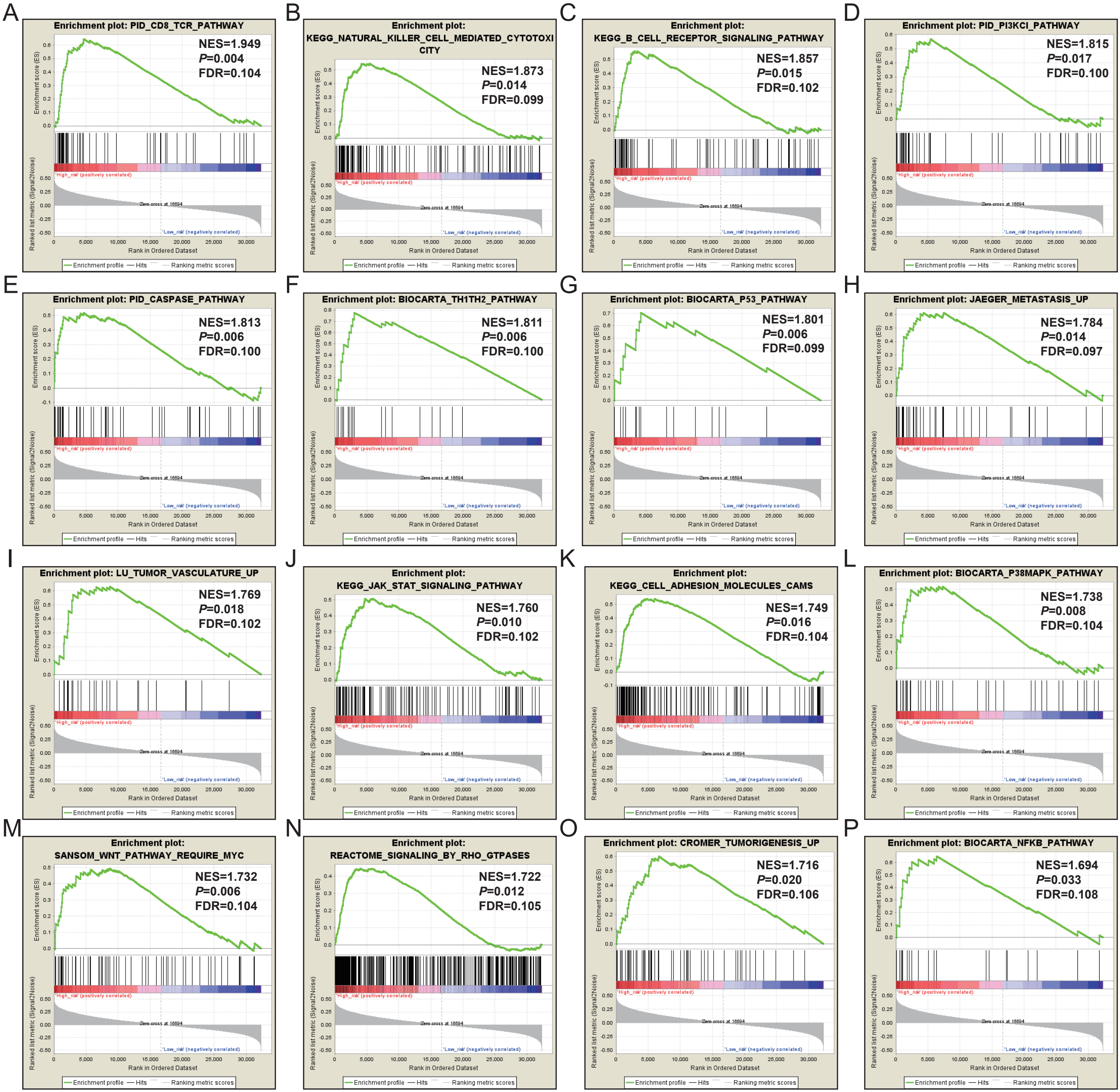

Figure S2. GSEA results using the c2 reference gene set. (A) CD8/TCR pathway; (B) natural killer cell medited cytotoxicity; (C) B cell receptor signaling pathway; (D) PI3KCI pathway; (E) caspase pathway; (F) TH1/TH2 pathway; (G) P53 pathway; (H) metastasis up; (I) tumor vasculature up; (J) JAK/STAT signaling pathway; (K) cell adhesion molecules CAMs; (L) P38/MAPK pathway; (M) Wnt pathway require Myc; (N) signaling by Rho gtpases ; (O) tumorigenesis up; (P) NF-κB pathway.
