# Supplementary material for: Integrative Analysis of a Novel Eleven-Small Nucleolar RNA Prognostic Signature in Patients With Lower Grade Glioma
Source: Front Oncol. 2021 Jun 7;11:650828. doi: 10.3389/fonc.2021.650828 (PMC8215672; doi:10.3389/fonc.2021.650828)
Supplement: Supplementary file 4 [file DataSheet_4.pdf]

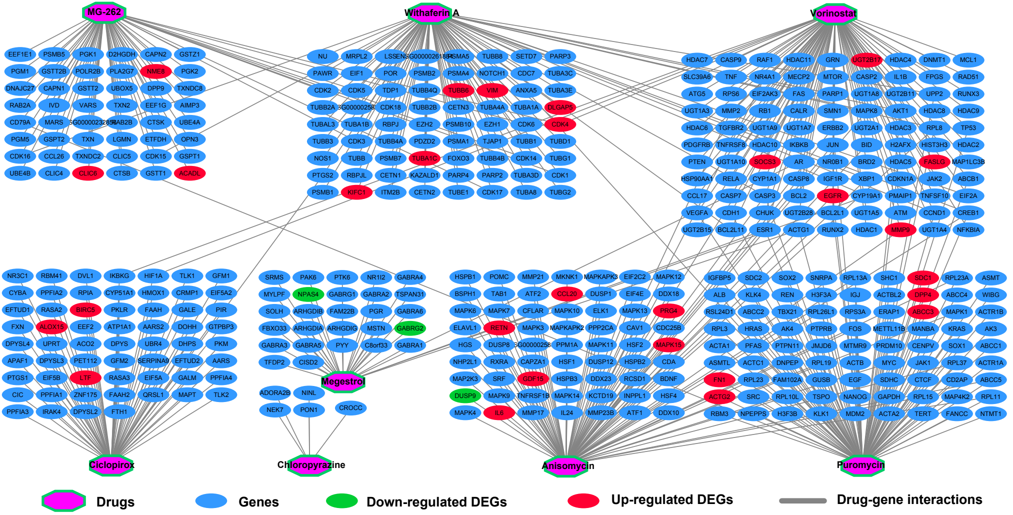


**Figure S4**. The drugs-genes interaction network of the targeted therapeutic drugs of snoRNAs prognostic signature.
