# Supplementary material for: Integrative Analysis of a Novel Eleven-Small Nucleolar RNA Prognostic Signature in Patients With Lower Grade Glioma
Source: Front Oncol. 2021 Jun 7;11:650828. doi: 10.3389/fonc.2021.650828 (PMC8215672; doi:10.3389/fonc.2021.650828)
Supplement: Supplementary file 5 [file DataSheet_5.pdf]

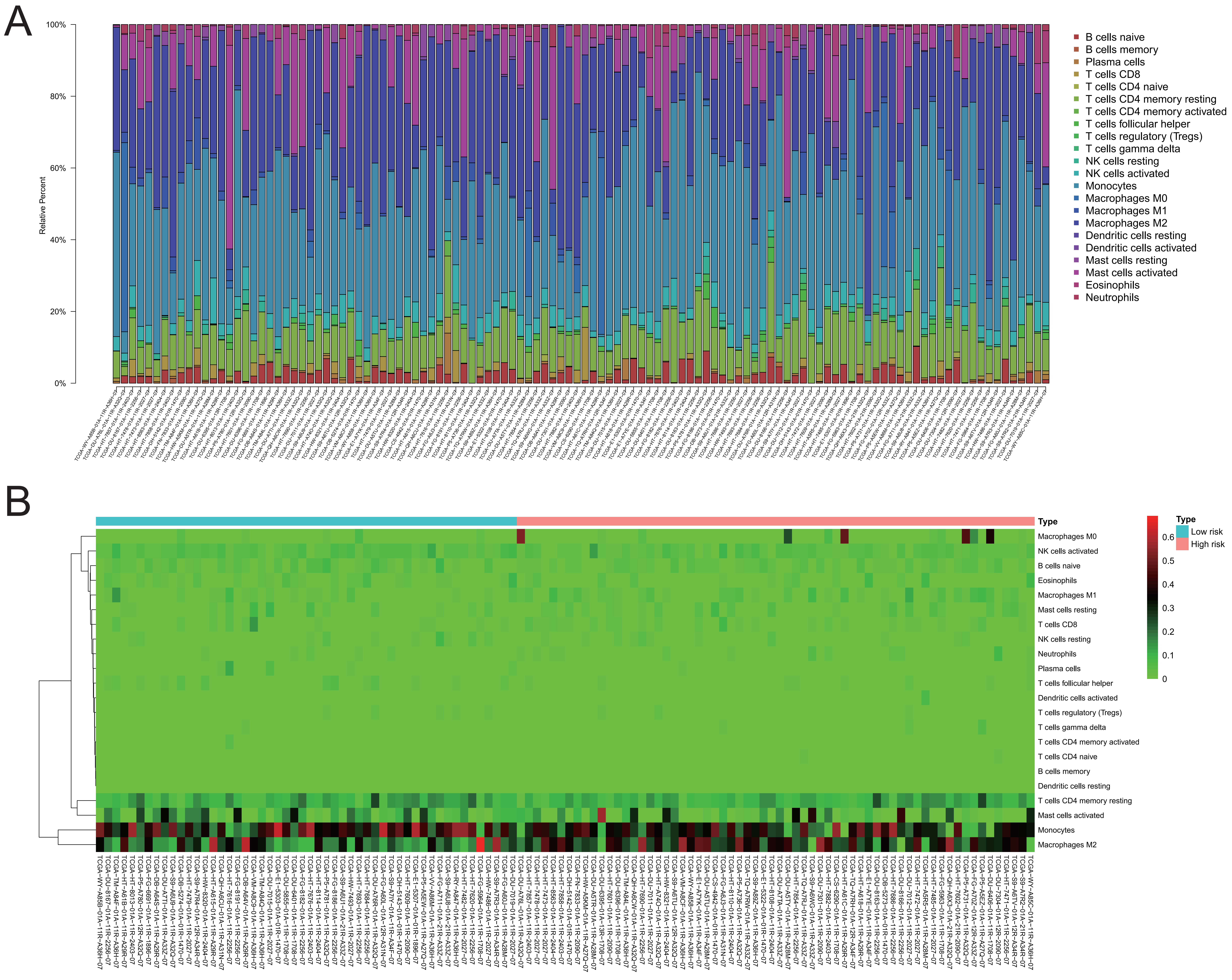

Figure S5. Immune infiltration analysis results of 22 immune cells in LGG tumor tissues. (A) Relative percent histogram of 22 immune cells in LGG tumor tissues; (B) Heatmap of 22 immune cells in LGG tumor tissues.
