# Supplementary material for: Integrative Analysis of a Novel Eleven-Small Nucleolar RNA Prognostic Signature in Patients With Lower Grade Glioma
Source: Front Oncol. 2021 Jun 7;11:650828. doi: 10.3389/fonc.2021.650828 (PMC8215672; doi:10.3389/fonc.2021.650828)
Supplement: Supplementary file 3 [file DataSheet_3.pdf]

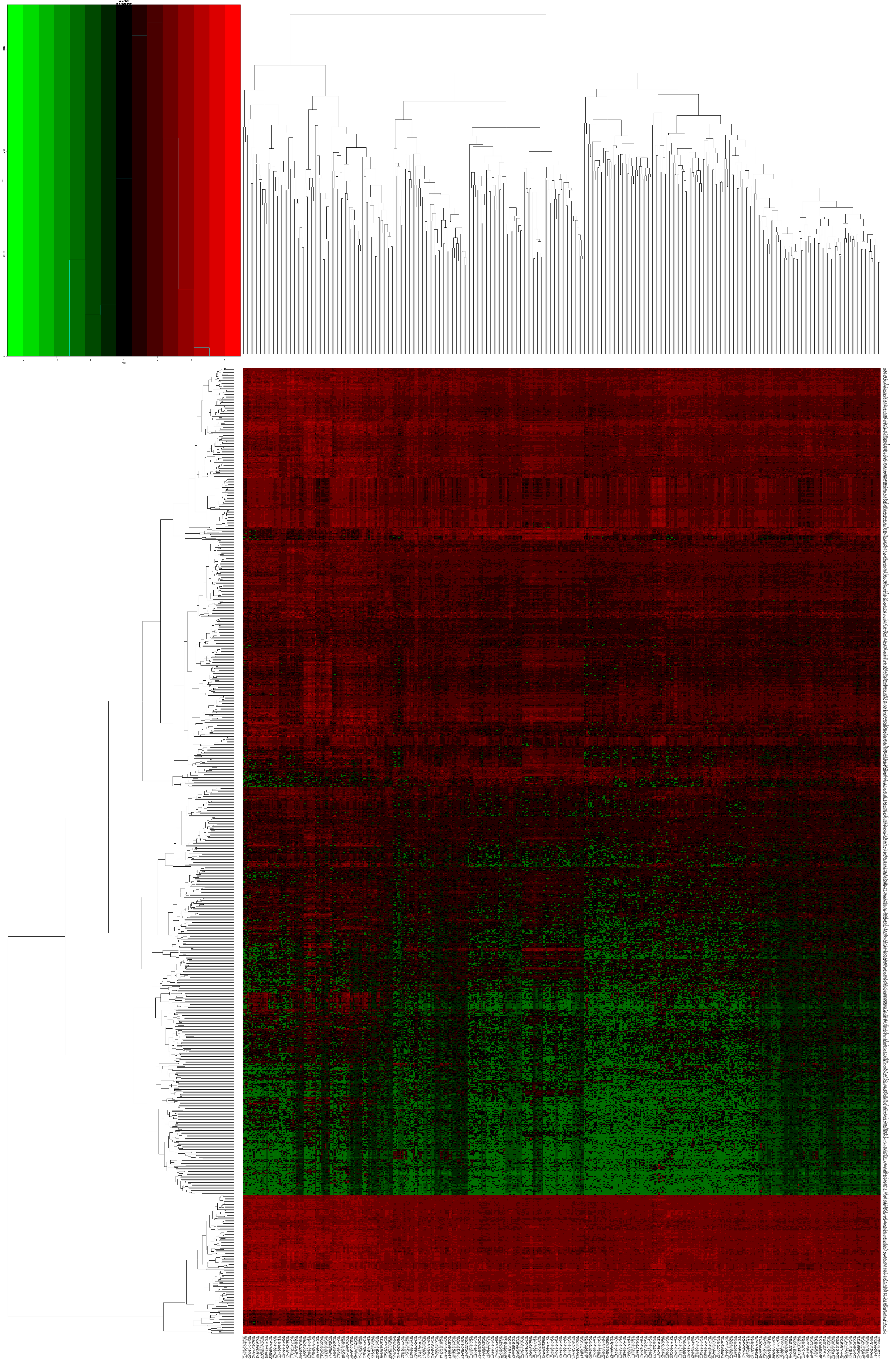

Figure S3. Heatmap of DEGs between high- and low-risk score phenotypes.
